# Rates of visual field progression in clinical glaucoma care

**DOI:** 10.1111/j.1755-3768.2012.02492.x

**Published:** 2012-10-16

**Authors:** Anders Heijl, Patricia Buchholz, Gunilla Norrgren, Boel Bengtsson

**Affiliations:** 1 Department of Clinical Sciences Malmö, Ophthalmology, Skåne University Hospital, Lund UniversityMalmö, Sweden; 2 Department for Medical and Pharmaceutical Issues, Regional Council KarlsruheKarlsruhe, Germany; 3 Allergan Ltd., Marlow InternationalThe Parkway, Marlow, Buckinghamshire, UK

**Keywords:** Glaucoma, progression, rate of progression, visual field

## Abstract

**Purpose:** To investigate rates of visual field progression and factors associated with progression rate in open-angle glaucoma in clinical glaucoma care.

**Methods:** We performed a retrospective chart review of all patients with manifest primary open-angle glaucoma (POAG) and pseudoexfoliation glaucoma (PEXG) followed ≥ 5 years with ≥5 SITA Standard fields. Exclusion criteria were minimal. Demographics, intraocular pressure values (IOP), treatment and treatment changes, and visual field (VF) data were recorded. VF progression rates were calculated as slopes of mean deviation (MD) over time.

**Results:** Five hundred and eighty-three patients were eligible. Three hundred and sixty-seven (62%) had POAG and 221 (38%) PEXG. Median MD at study start was −10.0 dB. Mean follow-up time was 7.8 years (SD ± 1.2); mean number of VF tests was 8.9 (SD ± 2.8). Progression rates varied very much among patients with a mean of −0.80 dB/year (SD ± 0.82; median rate, −0.62), and 5.6% of patients progressed at rates worse than −2.5 dB per year A negative slope of MD values was observed in 89% of patients. Mean IOP of all visits decreased over the study period from 20.15 to 18.10 mmHg. Higher age and mean IOP, and more intensive treatment were associated with more rapid progression, while PEXG and IOP variation were not, if treatment intensity was taken into account.

**Conclusion:** Rates of visual field progression in manifest glaucoma with field loss in ordinary clinical care were highly variable. Progression rates rapid enough to influence quality of life were common.

## Introduction

The large prospective clinical glaucoma trials and other studies have demonstrated that over longtime periods visual field defects progress in a large proportion of glaucoma patients and that progression rates vary very much among patients (Gliklich et al. 1989; Membrey et al. 2000; Eid et al. 2003; Zahari et al. 2006; Leske et al. 2007). Detailed data on rates of progression have been reported for untreated glaucoma, that is, the natural history of progression of glaucomatous visual field loss (VFL) in the prospective trials Collaborative Normal-Tension Glaucoma Study (CNTGS 1998) and Early Manifest Glaucoma Trial (EMGT; Leske et al. 1999; Anderson et al. 2001; Heijl et al. 2009). Data on rates of progression in ordinary clinical glaucoma care have been sparse until recently when important results have been reported from New York (Ahrlich et al. 2010; De Moraes et al. 2011; Forchheimer et al. 2011).

Rate of disease progression is one of the most important factors determining the risk of visual disability or blindness in glaucoma and several guidelines for glaucoma management recommend assessment of rate of progression in routine glaucoma care (European Glaucoma Society 2008; Heijl et al. 2010). Particularly against that background, it is desirable to learn more about rates of progression under ordinary clinical care and of risk factors associated with rate of progression.

In Sweden, most glaucoma care is delivered in the public sector, which is an unusual feature of eye care in other countries. As a matter of fact, most primary glaucoma care in our catchment area is delivered at our Department of Ophthalmology at Skåne University Hospital in Malmö. We therefore considered it meaningful to study glaucoma patients who receive glaucoma care at our department, because our glaucoma patients do not represent a selection of patients with particular characteristics, for example, patients with particularly aggressive or advanced disease.

Thus, the aim of this study was to study perimetric rates of progression and factors associated with progression in ordinary patients with open-angle glaucoma.

## Methods

The study was a retrospective review of patient charts.

### Inclusion and exclusion criteria, extracted data

We studied records of patients with diagnoses of primary open-angle glaucoma (POAG) or pseudoexfoliation (PEX) glaucoma. Potential patients were identified with the help of the computerized patient booking system of our hospital (Skåne University Hospital, Malmö, Sweden), comprising all visits during the last decades. Diagnoses are registered for all patients and visits, also for outpatient visits. The hospital provides primary glaucoma care for a majority of glaucoma patients (approximately ¾ of diagnosed patients) in the catchment area (population 300 000) in Southern Sweden. The study was performed in accordance with the declaration of Helsinki, and approval was obtained from the Ethical Review Board at Lund University, Sweden. Advertisements were published in local newspapers to allow glaucoma patients who had visited the department not to have data from their patient records included in the study.

Patient records were extracted for patients who had been followed for at least 5 years during the study period, from March 1996 to August 2005. The study period was selected in this way because the SITA Standard (Bengtsson et al. 1997) was introduced as the standard perimetric test in our department from March 1996, while data retrieval started in September 2005, and because patient records on paper were available for the whole study period.

In this study, glaucoma diagnosis required the presence of repeatable visual field defects in SITA Standard fields as defined by Glaucoma Hemifield Test results ‘Outside Normal Limits’. The diagnosis POAG also required open chamber angles, absence of exfoliation or pigment dispersion syndrome or signs of other secondary glaucomas. A glaucomatous eye was classified as having pseudoexfoliation glaucoma (PEXG) if there was any report of PEX syndrome in that eye at any visit.

Exclusion criteria were kept to a minimum: patients with other ophthalmic co-morbidity except cataract with serious influence on visual field results were not eligible. Other exclusion criteria were blindness in the study eye at study start, participation in the EMGT (*n* = 180; Leske et al. 1999) or that the patient did not want to contribute with data in the chart review (*n* = 1).

Eligibility required continuous follow-up for ≥5 years and that ≥5 Humphrey SITA Standard visual field tests were available.

One study eye was identified for each patient. The study eye was the eye with VFL or, if both eyes had VFL, the eye with the largest VFL defined by the global perimetric mean deviation (MD) index.

Clinical parameters collected from patient charts and reported in this paper were age; gender; and – from study eyes – PEX status, MD values for all perimetric tests performed during the study period, medication and changes in medication, incisional and laser (ALT) surgeries, and concomitant eye disease.

### Statistical analysis

Descriptive analyses were performed on demographics, follow-up time, baseline MD and on intraocular pressure (IOP), IOP variation defined as the range between the highest and lowest IOP values measured during the study period, and MD values over time. Study start for a patient was defined as the first visit that contributed records data from the patient. For patients in whom follow-up started before 1996, the study start was thus usually in 1996, that is, when the first SITA Standard fields were obtained. For patients diagnosed after 1996, the baseline usually was the first visit after diagnosis.

For each study eye, we calculated visual field progression rate using linear regression analysis of perimetric MD values over time, where rate of progression is the slope expressed in dB/year. Reliability criteria, that is, fixation losses, false positive or false negative answers, were not taken into account, and only a few obviously artefactual fields, for example, clover leaf fields or similar extreme outliers, were excluded from analysis. We also calculated mean IOP over all study visits and IOP variation.

Changes in drug treatment were registered and compounded into a drug change score. Each change added one to this score, so that a patient who was on the same drug treatment during the study period received a score of 0, while a patient who was switched to another drug or in whom one drug was added, got a score of 1, and a patient who encountered three changes received a score of 3.

We studied factors that we assumed might be associated with progression rate. In a first multiple linear regression analysis, we included age, gender, mean IOP, IOP variation, presence of exfoliation syndrome (PEX) and MD at study start. All parameters that were not normally distributed (IOP range, MD, drug change score) were then divided by median split.

As treatment may change IOP range, we subsequently performed a second multivariate analysis adding treatment variables: the drug treatment score, incisional surgery and ALT.

In a third multivariate analysis, we removed all variables that did not reach the p < 0.10 significance level in the second analysis.

All statistical analyses were performed using spss v. 19.0 (IBM SPSS, Chicago, IL, USA).

## Results

All eligible patients (*n* = 583) were included in the analysis. Mean age at start was 71.4 years (min 31; max 95). The majority of patients, 367 (63%), were female.

**Fig 1:**
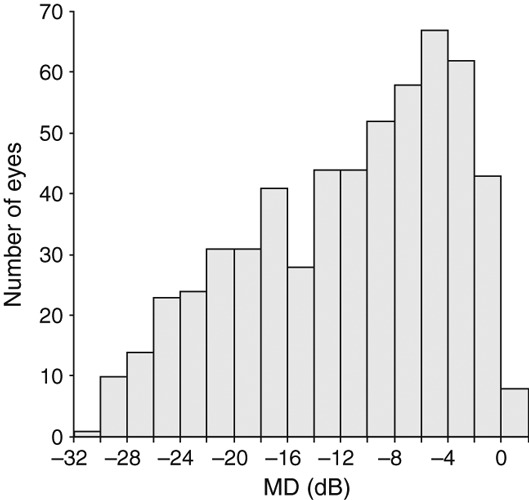
Mean deviation (MD) at the beginning of the follow-up period. The whole spectrum of glaucomatous field loss is covered. The median MD (−10.0 dB) corresponds to moderately advanced glaucoma.

Three hundred and sixty-seven eyes (62%) had POAG, while 221 (38%) had PEX glaucoma. The range of disease severity was large (Fig. 1). Expressed as MD values in the study eye at study start, severity ranged from −30.4 to +1.6 dB; the median MD corresponded to moderately advanced glaucoma, −10.0 dB (Mills et al. 2006).

Mean follow-up time for the study period was 7.8 years (SD ± 1.2; range, 5.0–9.6 years). The mean number of visual field tests per eye was 8.9 (SD ± 2.8; range, 5–25).

**Fig 2:**
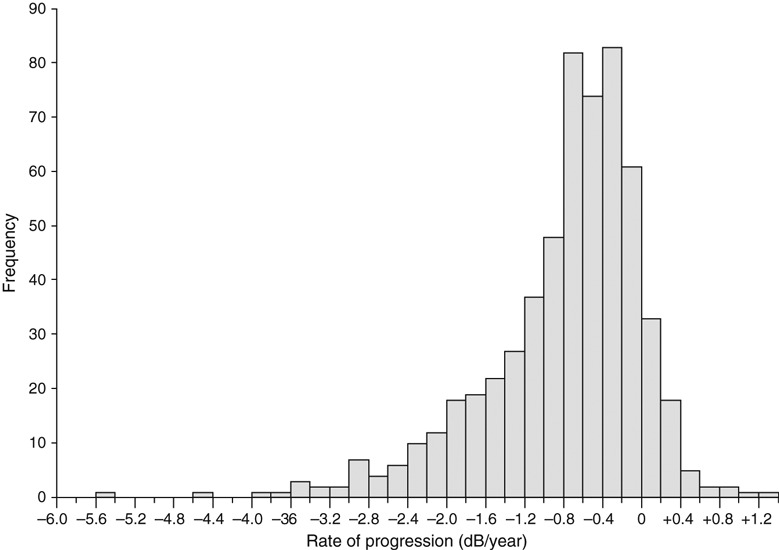
Rates of progression expressed in dB/year. The distribution is wide and has a negative tail of rapidly progressing eyes.

**Fig 3:**
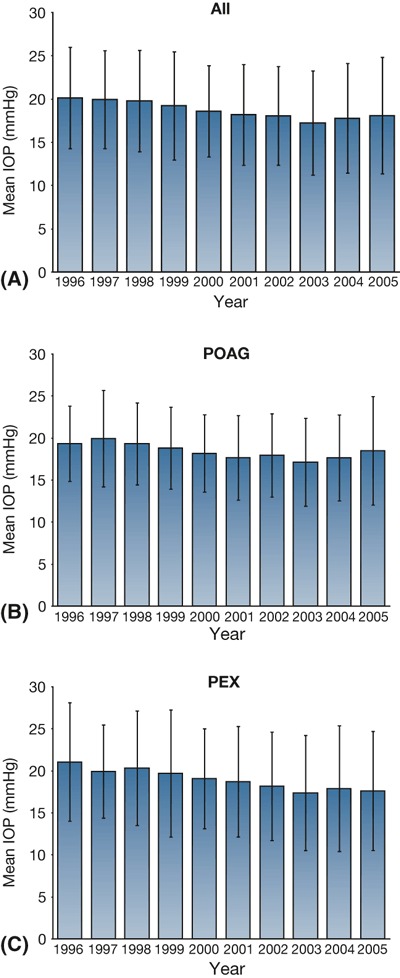
Mean intraocular pressure (IOP) over time ±1 standard deviation for all eyes (A), for the group of eyes with primary open-angle glaucoma (B) and exfoliation glaucoma (C). IOP decreased slowly over time.

Progression rates are clear from Fig. 2. The mean MD slope was −0.80 dB/year (SD ± 0.82, median, −0.62), and slopes ranged from −5.58 to +1.24 dB/year (Fig. 3) with a negatively skewed distribution. Eighty-nine percentage of slopes were negative; 60% statistically significant at the p < 0.05 level.

### Intraocular pressure

Mean IOP and SD for each study year are shown in Fig. 3 for all eyes and for eyes with POAG and PEX glaucoma. Mean IOP in the whole cohort decreased from 20.15 mmHg in 1996 to 18.10 mmHg in 2005 (Fig. 3). IOP variation (highest – lowest recorded IOP) varied among study eyes from 2 to 61 mmHg, with a median of 13.

### Medications and surgery

The median number of additions and changes in medication was 3; minimum 0 and maximum 30. Argon laser trabeculoplasty was performed in 179 eyes, once in 127 eyes, twice in 48 and three times in four eyes. Sixty eyes underwent incisional surgeries (all trabeculectomies), all once only.

### Factors associated with progression

The results of the first multiple linear regression analysis are shown in Table 1. Older age, higher mean IOP and IOP range were all associated with significantly faster progression (more negative slopes), while gender and presence of pseudoexfoliation syndrome were not. More field loss at start of the follow-up period was associated with slower progression, possibly because of truncation (cf. Discussion).

**Table 1:** Results of multivariate analysis of factors associated with rate of progression not including treatment parameters.

Variable	Reference	Slope	Significance
Age	N/A	−0.019	0.000
Mean IOP	N/A	−0.036	0.001
IOP range*	<12 mmHg	−0.256	0.000
MD*	Worse than −10.03 dB	−0.175	0.009
PEX syndrome†	No	−0.071	0.206
Gender	Male	0.078	0.250

IOP, intraocular pressure; MD, mean deviation.

* Divided by median split.

† Yes/no.

When the treatment parameters medications, ALT and trabeculectomies were added to the multivariate analysis, IOP range was no longer statistically significant (p = 0.101) while mean IOP (p = 0.033), age (p = 0.000) and MD remained significant. As expected, this analysis also showed that changes in medication, ALTs and trabeculectomies were more common in eyes with steeper negative slopes (drug change score p = 0.003; ALT p = 0.041; trabeculectomies p = 0.040).

The results of the third multivariate analysis including only factors significant at the p < 0.1 level in the second multivariate analysis are shown in Table 2. Age, mean IOP, MD at study start, ALT and trabeculectomies were all significantly associated with more rapid progression.

**Table 2:** Results of multivariate analysis of factors associated with rate of progression when including treatment parameters.

Variable	Reference	Slope	Significance
Age	N/A	−0.021	0.000
Mean IOP	N/A	−0.028	0.011
MD*	Worse than −10.03 dB	−0.188	0.004
ALT†	No	−0.182	0.021
Trabeculectomies†	No	−0.283	0.012

IOP, intraocular pressure; MD, mean deviation.

* Divided by median split.

† Yes/no.

## Discussion

In this large group of patients treated for open-angle glaucoma, a majority progressed during a mean follow-up time of 7.8 years.

Rate of progression varied very much among patients; the median rate of progression corresponded to going from a normal to a blind field in approximately 50 years, but a considerable minority progressed much faster. Fast progression, for example >1.0 dB/year, was relatively common (Fig. 2). Progression at such rates is clearly important for quality of life. In 10–15 years, eyes progressing at such rapid rates would go from early glaucoma to advanced glaucoma or from moderate to severe disease (Mills et al. 2006).

Study results confirmed mean IOP as risk factors for glaucoma progression. In initial analyses, IOP range was also a significant risk factor in first multivariate analyses, but when factors indicating treatment and treatment changes were added to the analyses, the IOP range was no longer significant.

This study has strengths and weaknesses. Among the strengths is the large number of patients analysed. The data are also quite representative for all treated glaucoma patients in the catchment area. This is attributable to the unusual structure of Swedish ophthalmic care, where a single university hospital site can provide most primary glaucoma care in a large catchment area. In most other EU countries, this type of data is not obtainable as glaucoma patients have access to multiple health care providers and receive treatment in a variety of settings: office-based private practices, private clinics or state-owned clinics/hospitals. Another strength is that most patients came to regularly scheduled appointments, which led to a relatively large number of fields collected over a time interval long enough for reasonable assessments of rate of progression. The fields were also always obtained with the same SITA Standard 30-2 test, eliminating errors caused by different threshold characteristics among tests (Heijl et al. 2000).

This is a retrospective study. This is a weakness because much data, for example, on systemic blood pressure and cardiac disease are missing, which makes it impossible to analyse whether such systemic health parameters were associated with glaucoma progression rate. At the same time, the retrospective study design can be a considered strength, resulting in data that represent ordinary routine clinical care. We consider it likely that with a prospective study design, eyes with higher progression rates would have received more drastic changes of treatment, including surgery, that might have resulted in lower IOP values and somewhat lower progression rates and that adherence might have been higher than in ordinary glaucoma care.

Treatment intensity and target pressures may of course differ between centres and will influence rate of progression. Even small differences in IOP may make a difference. Thus, several of the large prospective trials of glaucoma and ocular hypertension have shown that the risk of progression may on the average decrease by 10% or more for each mmHg of IOP reduction (Gordon et al. 2002; Leske et al. 2003; Miglior et al. 2007; Chauhan et al. 2008). Even if percentage reduction of risk is not identical to reduction of rate of progression, they are closely related. The Malmö department did not have a strict, written care programme for glaucoma, and over 20 physicians were involved in the glaucoma care. Experience varied among physicians most of whom were not glaucoma specialists.

IOP levels on treatment reflect the level of treatment intensity. It is therefore of interest to compare the IOP levels in the current study with other published figures. In the current study, mean IOP was just over 20 mmHg in 1996 and decreased by 2 mmHg during the study period. These IOP values are similar to those found in other chart analyses performed in Europe during the same time period. In a consecutively recruited retrospective chart analysis in Sweden and France of several hundred patients with open-angle glaucoma and ocular hypertension, mean IOP over time in treated patients was 18.2 ± 4.2 mmHg, 18.9 ± 4.9 in the Swedish group (Lindblom et al. 2006). This is very similar to the results of the current results, particularly because our means include some untreated IOP values in newly diagnosed patients. A retrospective medical record analysis in several academic and office-based centres in Sweden and the United States had had very similar IOP levels at study end, in USA 18.4 ± 4.3 mmHg; Sweden 18.8 ± 5.3 mmHg (Kobelt-Nguyen et al. 1998). In a Dutch study of 500 representative patients with glaucoma and ocular hypertension, the mean (±SD) IOP in the 355 patients with glaucoma was 20.9 ± 6.9 mmHg (Oostenbrink et al. 2001). Thus, IOP levels seem not to have differed much among the current and other published studies.

There are some recent comparable studies that report rates of visual field progression, particularly in clinical settings. Several of those come from the same research group in New York. The closest comparison may be a study by De Moraes et al. (2011). This paper focuses on risk factors for progression but also reports global rates visual field progression expressed as MD loss/year. The study has several similarities with ours: it is of very similar size, duration, and also number of fields, and the study population is mainly white, with a majority of women. Their patient group was quite different, however. The authors emphasize that they represent a tertiary referral centre. They included a more mixed patient group including narrow angle and juvenile glaucoma, and the patients were on the average 6.5 years younger at baseline, and mean MD values were better.

As it is known that the risk of progression in many trials decreases by 10–19% per mmHg of IOP reduction (Gordon et al. 2002; Leske et al. 2003; Miglior et al. 2007; Chauhan et al. 2008), it might be permissible to assume that rates of progression are similarly influenced by IOP, particularly because risk calculations in trials often are based on analyses that are based on time to progression. If mean IOP in our cohort had been at the same level as that in de Moraes’ group, and assuming that the risk/rate reduction per mmHg is similar to that discussed above, it is reasonable to assume that our progression rates had been 30–40% slower and thus quite similar to those reported by Moraes. We therefore feel that the results of the two studies are in agreement. The effect of age on progression has been estimated, for example in a report from the Canadian glaucoma study, where the hazard ratio was 1.04 per year of increasing age (Chauhan et al. 2010, 2008). Here, it was 1.09 per year. A study by Forchheimer et al. (2011) reports progression rates that are similar to those of Moraes in a similar group of patients. The aim of the Forchheimer’s study was to study the influence of baseline perimetric status, and it is likely that there is considerable overlap in the patient groups studied by de Moraes and Forchheimer.

We chose to include the worse eye in patients with bilateral glaucoma, mostly to be sure that study eyes really had manifest glaucoma. This may have increased the risk of truncation (floor effects), when visual fields defects become more and more advanced. All studies like the present one have some problems with floor effects, and, therefore, reported mean rates of progression should be regarded as minimum estimates.

The Canadian Glaucoma Study also reported rates of progression (Chauhan et al. 2010). Those rates were much lower than ours, with a mean rate in 45 progressing patients of −0.35 dB/year and a slightly positive change (+0.05 dB/year) in 153 nonprogressing patients. Our results are not at all in line with these Canadian results, but the differences between the studies were large. Thus, the Canadian patients were approximately 8 years younger than the Swedish patients, their mean IOP was much lower at 14.8 mmHg, the proportion of PEXG was also much lower, and the patients had considerably earlier glaucoma (study eligibility required MD values better than −10 dB). Another factor that may have contributed to the considerably better progression rates in the Canadian study was that these patients were followed in a well-organized, prospective study. Patients in such studies are generally assumed to have considerably better adherence to prescribed therapy than patients in routine medical care. It is tempting to guess that this might be the main factor, because mean age and mean IOP are in fact quite similar in the Canadian study and the studies from New York (De Moraes et al. 2011; Forchheimer et al. 2011), while progression rates are very different.

It is of some interest to also compare our observed progression rates, with such rates in untreated glaucoma. Two studies provide such natural history progression rates, the CNTGS and the EMGT (Anderson et al. 2001; Heijl et al. 2009). Reported mean rates in normal-tension glaucoma eyes are similar in CNTGS and EMGT, approximately 0.4 dB/year. This is less than the median rate in the current study of treated eyes. Untreated rates differ much between groups of glaucoma patients, however. The mean rate in EMGT was 1.08 dB/year, in high tension glaucoma it was 1.31 dB/year and in PEX glaucoma 3.13 dB/year. It is therefore clear that despite the fact that mean and median progression rates in the current study were not slow, they were considerably slower than in untreated patients from the same population.

Reflecting on the generalizability of the results, we must notice that all data were collected at a single site with mostly Caucasian patients and a rather high percentage of exfoliation glaucoma. We still believe that the patient population is reasonably representative of glaucoma patients in this part of Europe. PEXG is common in Malmö, but also in the neighbouring Nordic countries as in many other parts of the world, for example, Greece, Russia, Turkey, India, parts of Africa (Ritch 2001; Ritch & Schlotzer-Schrehardt 2001). Stage of disease could be of importance. In Sweden, asymptomatic individuals only rarely see ophthalmologists for check-ups; individuals with a positive family history of glaucoma may be an exception. Glasses are often dispensed by opticians with limited capability of detecting glaucoma by means other than with tonometry, which is often performed by opticians in customers over the age of 50. As a result, clinical diagnoses of glaucoma are often made late (Grødum et al. 2002). Mean MD at study start was also worse (−10.0 dB) in this study than in several other large studies reporting rates of progression (Ahrlich et al. 2010; Chauhan et al. 2010; De Moraes et al. 2011; Forchheimer et al. 2011).

Our analyses of factors associated with progression confirm some other risk factors that have been identified in controlled trials. IOP was found to be a risk factor in the EMGT (Leske et al. 2003, 2007), AGIS (The AGIS Investigators 2000), CIGTS (Lichter et al. 2001) and Canadian Glaucoma Study (Chauhan et al. 2008), while EMGT and AGIS (The AGIS Investigators 2002) also found age to be significant factors. Age was a significant factor also in the Canadian Glaucoma Study (Chauhan et al. 2008). EMGT results additionally defined presence of PEX as a risk factor (Leske et al. 2007).

Several treatment trials have indicated higher risk of progression in eyes with worse MD values (The AGIS Investigators 2002, Leske et al. 2007). In the current study, worse visual field status was not a risk factor for more rapid progression, but the opposite. Worse baseline field status was associated with slower measured progression. It is likely that this was attributable to truncation; a visual field with very advance cannot progress as much as a field with smaller defects. A recent study by Forchheimer et al. (2011) found no difference in progression rates depending on MD after correcting for IOP.

The appearance and disappearance of IOP range as a risk factor is very interesting, however, considering the widely divided opinions on this matter (Singh & Shrivastava 2009). In our initial analyses, which did not take treatment changes into account, larger IOP range was a significant risk factor, but when factors flagging treatment intensity were included in the analysis, the significance of range disappeared. Instead, the results showed that treatment intensity was indeed positively correlated with worse progression rates. The significance when treatment changes were unaccounted for is in agreement with other studies, when results have been analysed without correcting for such changes (Nouri-Mahdavi et al. 2004; Bengtsson et al. 2007; Singh & Shrivastava 2009). Also, the AGIS investigators found that IOP variation was less important and only significant in eyes with low IOP levels, when they limited their analysis to eyes that had only one intervention (Caprioli & Coleman 2008). We have previously shown that if treatment changes are accounted for apparent significance of IOP variations may disappear, while mean IOP remained a highly significant factor (Bengtsson et al. 2007).

Our drug change score can be criticized for being arbitrary and not differentiating between changes initiated to further decrease IOP or for other reasons, for example, encountered side effects. In a retrospective chart analysis like the present one, it is not always clear why a prescription has been changed, however, but the change itself is clearly documented. This is one reason why we decided to simply add management changes. Another advantage is that this approach leaves no room for subjective biases when data are extracted and analysed.

The results support the notion that this study like any other study that allows treatment changes is not really suited to investigate the effect IOP variation. If clinical care is delivered in an optimal way, one would expect treating physicians to intensify treatment in progressing patients, thus producing a larger IOP variation in eyes with more rapid progression. Our analyses showed that this was indeed the case.

Thus, we found that rates of visual field progression in manifest glaucoma with field loss in ordinary clinical care were highly variable among patients. Most study eyes progressed, and progression rates that were rapid enough to influence quality of life over a 10–15 year period were common. The risk factors for progression were higher mean IOP and older age. Presence of pseudoexfoliations was not a risk factor in a multivariate analysis, nor was IOP range, if treatment factors were included in the analyses.
